# Ancient bacteria–amoeba relationships and pathogenic animal bacteria

**DOI:** 10.1371/journal.pbio.2002460

**Published:** 2017-05-02

**Authors:** Joan E. Strassmann, Longfei Shu

**Affiliations:** Department of Biology, Washington University in St. Louis, St. Louis, Missouri, United States of America

## Abstract

Long before bacteria infected humans, they infected amoebas, which remain a potentially important reservoir for human disease. Diverse soil amoebas including *Dictyostelium* and *Acanthamoeba* can host intracellular bacteria. Though the internal environment of free-living amoebas is similar in many ways to that of mammalian macrophages, they differ in a number of important ways, including temperature. A new study in *PLOS Biology* by Taylor-Mulneix et al. demonstrates that *Bordetella bronchiseptica* has two different gene suites that are activated depending on whether the bacterium finds itself in a hot mammalian or cool amoeba host environment. This study specifically shows that *B*. *bronchiseptica* not only inhabits amoebas but can persist and multiply through the social stage of an amoeba host, *Dictyostelium discoideum*.

## Environmental amoebas came before animals as hosts to bacteria

The bacteria that most concern us are those that make us sick, but we are sometimes so preoccupied with our battle with them that we forget they have been waging a much longer war. More than a billion (10^9^) years before the first animals, bacteria were evolving strategies first to resist being killed by protozoan predators and then to actually infect their former predators [1]. These strategies are likely to have laid the groundwork for the later evolution of animal–bacteria interactions, so understanding how they function provides an essential context for understanding modern-day bacterial pathogens in humans. This is particularly true for the bacteria that invade animals through macrophages [2]. Further, environmental amoebas are still ubiquitous in modern soil and water, so they may act as important reservoirs from which emerging human diseases can arise [3]. Many amoebas, including *Acanthamoeba castellanii*, *D*. *discoideum*, *Hartmannella vermiformis*, and *Naegleria gruberi*, have been found to harbor bacteria [4]. Bacteria that can defeat amoebas’ defenses gain a refuge in which to proliferate, where they are protected from hostile external conditions by their unwitting hosts [5–8].

It is worth pointing out that amoebas do not fall into a monophyletic group but instead share a life form and a diet based on phagocytosis. The bacteria that can evade amoeba defenses are called amoeba-resistant bacteria [3,4]. In these amoebas, resistant bacteria can survive, proliferate, and be protected in adverse situations, particularly when the host amoeba forms a hardy cyst with the bacteria inside.

**Glossary**

**Amoeba-resistant bacteria**: Bacteria that have evolved to resist being killed by free-living amoebas.

**Bacterial secretion system**: The mechanisms by which bacterial pathogens evolved to export various virulence factors across the phospholipid membrane and cell envelope.

**Ejectosome**: A peripheral cellular organelle responsible for ejecting cytosolic bacteria from the cell without lysing that cell.

**Fruiting body**: A multicellular structure on which spore-producing structures are borne.

**Free-living amoebas**: Widely distributed protozoa that have the ability to alter their shape and feed on bacteria, algae, fungi, and small organic particles.

**Lysosome**: A membrane-bound organelle that contains hydrolytic enzymes that can break down biomolecules.

**Phagocytosis**: The process by which a cell engulfs a solid particle to form an internal compartment known as a phagosome.

**Phagosome**: A vacuole formed around a particle engulfed by phagocytosis.

**Symbiosis**: A relationship between individuals of different species that live closely together.

**Two-component regulatory system**: One kind of mechanism of signal transduction that allows organisms to sense and respond to a changing environment.

**Spore**: A unit of sexual or asexual reproduction that is able to disperse and survive in unfavorable conditions.

**Virulence factor**: Molecules produced by pathogens that can increase their fitness in interactions with the host.

## Survival strategies of intracellular bacteria within amoebas

Entry of bacteria into amoebas is simple because amoebas eat bacteria. Amoebas normally engulf food bacteria by phagocytosis and kill them inside the phagosome, where ingested bacteria are confronted with acidification, oxidative stress, nutrient deprivation, and various antimicrobial small molecules [2] [9,10]. Amoeba grazing has been suggested to be one of the major forces shaping bacterial abundance and diversity [11]. However, some bacteria have developed strategies to survive phagocytosis by amoebas and are able to exploit host cell resources. Bacteria like *Legionella pneumophila* that remain in the vacuole of macrophages in humans are perhaps the best-studied bacteria that infect humans and amoebas, but they are by no means the only ones (Table 1) [12,13].

**Table 1:** List of human pathogens that are found in free-living amoebas. These bacteria are isolated from various amoeba hosts and have different lifestyles [8,14–16]. They have evolved sophisticated ways to export various virulence factors across their bacterial inner and sometimes outer membrane (in gram-negative bacteria), as well as through the host plasma membrane or phagosomal membrane, by using diverse secretion systems [17,18].

*Bacteria*	Amoeba hosts	Location in amoebas	Bacterial secretion systems known to be present	Human diseases
***β proteobacteria***				
*Burkholderia cepacia*	Acanthamoeba	Extracellular	Type III secretion system; type VI secretion system	Pneumonia
*Bu*. *pseudomallei*	Acanthamoeba	Extracellular	Type III secretion system; type VI secretion system	Melioidosis
*Burkholderia* spp.	Dictyostelium	Facultative intracellular	Unknown	Unknown
***γ proteobacteria***				
*Coxiella burnetii*	Acanthamoeba	Obligate intracellular	Dot/Icm type IVB secretion system	Q fever
*Escherichia coli O157*	Acanthamoeba	Extracellular	Type III secretion system; Tat secretion pathway	Hemorrhagic diarrhea; kidney failure
*Francisella tularensis*	Acanthamoeba	Facultative intracellular	Type VI secretion system	Tularemia
*L*. *pneumophila*	Various amoebas	Facultative intracellular	Type II secretion system; type IV secretion system; Tat secretion pathway	Legionnaires disease
*L*. *anisa*	Acanthamoeba	Facultative intracellular	Unknown	Pontiac fever; Legionnaires disease
*Pseudomonas aeruginosa*	Acanthamoeba	Extracellular	Tat secretion pathway; Type VI secretion system	Infect human cells
*Vibrio cholerae*	Acanthamoeba, Naegleria	Extracellular	Type I secretion system; type II secretion system; type VI secretion system	Cholera
***ɛ proteobacteria***				
*Helicobacter pylori*	Acanthamoeba	Facultative intracellular	Type IV secretion system	Asymptomatic disease
***Chlamydia***				
*Chlamydophila pneumoniae*	Acanthamoeba	Obligate intracellular	Type III secretion system	Pneumonia
*Neochlamydia hartmanellae*	Hartmannella	Obligate intracellular	Type III secretion system	Infect human cells
*Parachlamydia acanthamoebae*	Acanthamoeba	Obligate intracellular	Type III secretion system	Infect human cells
*Simkania negevensis*	Acanthamoeba	Obligate intracellular	Type III secretion system	Chronic obstructive pulmonary disease
***Bacilli***				
*Listeria monocytogenes*	Acanthamoeba	Facultative intracellular	Type VII secretion system	Listeriosis
*Bacillus anthracis*	Acanthamoeba	Obligate intracellular	Type IV secretion system	Anthrax
***Actinobacteria***				
*Mycobacterium leprae*	Acanthamoeba	Obligate intracellular	Type VII secretion system	Leprosy
*M*. *avium*	Acanthamoeba	Facultative intracellular	Type VII secretion system	*Mycobacterium avium-intracellulare* infection
*M*. *marinum*	Acanthamoeba	Facultative intracellular	Type VII secretion system	Opportunistic infections; aquarium granuloma
*M*. *ulcerans*	Acanthamoeba	Facultative intracellular	Type VII secretion system	Buruli ulcer

**Fig 1:**
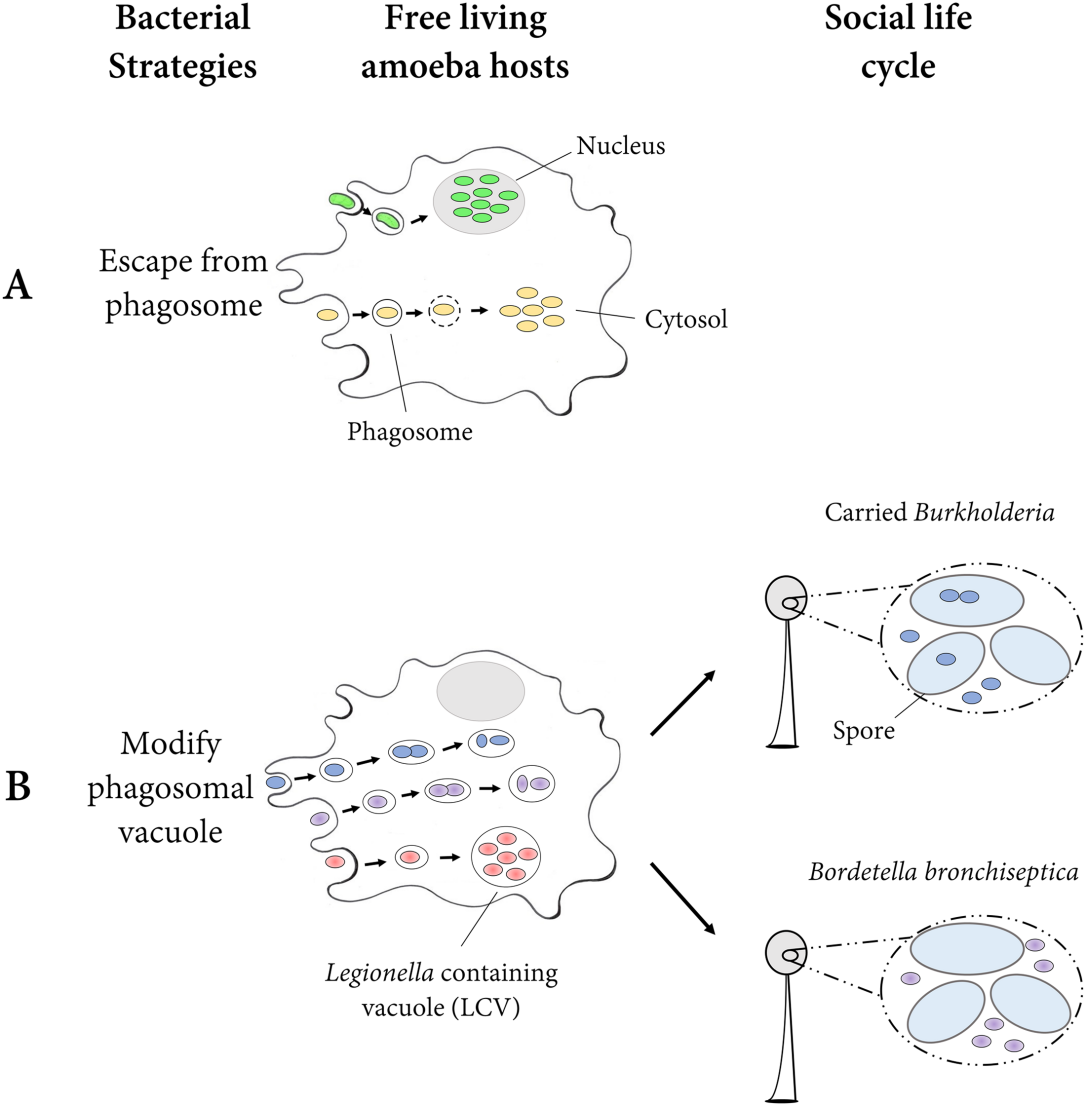
Diagram of survival strategies of intracellular bacteria within amoebas. The figure represents two general strategies that intracellular bacteria deploy to survive within amoebas. They can escape from the phagosome (Fig 1A) or stay within the phagosomal vacuole but modify it (Fig 1B). Green, intranuclear bacteria; yellow, bacteria that escape into the cytosol; blue, carried *Burkholderia*; purple, *B*. *bronchiseptica*; red, *L*. *pneumophila*.

The most obvious strategy to avoid being killed by the amoeba host is to escape from its vacuole into the cytosol of the amoeba (Fig 1A). For example, *M*. *marinum* and *M*. *tuberculosis* have evolved this ability (Fig 1A, yellow). This process requires the mycobacterial type VII secretion system ESX-1 [12]. In addition, both *M*. *marinum* and *M*. *tuberculosis* can eject from the cell through an F-actin structure called an ejectosome and then spread from cell to cell [19,20].

In general, the cytosol is considered to be favorable for bacterial growth because it provides nutrients and is isolated from the host immune system [21]. Therefore, it is an ideal place for bacteria to thrive after escaping from the phagosome. Some intracellular pathogens can invade a more unusual intracellular niche: the eukaryotic nucleus (Fig 1A, green). This has been reported in the free-living amoebas—for example, a bacterium called strain Pn in Chlamydiae was found in nuclei of *N*. *clarki* [22]. A bacterium tentatively called “*Candidatus* Nucleicultrix amoepiphila” and distantly related to the *Rickettsiales* was found in nuclei of *Hartmannella* sp. [23]. Apparently, these two evolved the intranuclear habit independently.

The second strategy bacteria employ is to stay within the phagosomal vacuole but to subvert its antimicrobial mechanisms (Fig 1B). These subversion techniques include preventing phagosome-lysosome fusion, modulating phagosomal pH, damaging phagosomal membranes, and/or quenching oxidative bursts [5]. Intracellular pathogens use a combination of these approaches. For instance, *L*. *pneumophila* (Fig 1B, red) has evolved a complex system that allows the bacteria to hijack the phagocytic vacuole [24]. It evades the endocytic pathway and the subsequent phagosome-lysosome fusion, delays its acidification, and establishes a safe intracellular niche called a *Legionella*-containing vacuole (LCV), which allows intracellular replication [24,25]. Further studies suggest that *L*. *pneumophila* uses the Icm/Dot type IV secretion system (T4SS) and the Lsp type II secretion system (T2SS) to avoid death and to exploit host resources [24,26]. Other bacteria use similar strategies [12].

The well-studied amoeba *D*. *discoideum* adds another wrinkle to the story of amoeba–bacteria interactions. This social amoeba in the Amoebozoa and others in its family behave like other soil amoebas most of the time, eating bacteria and dividing by binary fission. But when they cease to find sufficient food bacteria, the amoebas aggregate by the tens of thousands into a multicellular slug that moves towards heat and light [27]. Ultimately, this slug forms a fruiting body in which about 20% of cells (formerly independent amoebas) die to form a sturdy stalk, and the remaining cells form hardy spores atop the stalk, where they are more likely to be transported [27,28].

Bacteria can exploit this amoeba [29,30]. Some bacteria can also remain inside the spores through the social cycle. *Burkhoderia* near *fungorum* is one such bacterium (Fig 1B). In fact, this and other strains of *Burkholderia* so change the phagosome machinery that *D*. *discoideum* infected with them can also carry food bacteria, which would otherwise be digested (Fig 1B, blue) [31–34]. These amoeba clones are called farmers because they can seed and harvest their crops in new environments [34].

Overall, the majority of intracellular pathogens of amoebas occupy phagosomal vacuoles, while only some are able to escape the phagosome [5]. This is possibly because specialized mechanisms are needed to escape from the phagosome [5,21]. There is no clear relationship between the type of survival strategies and whether the microbe is an obligate or facultative intracellular pathogen [5].

## Interactions between *B*. *bronchiseptica* and amoebas

We began this piece by noting that amoebas antedated animals on the planet by more than a billion years. If bacteria began their infectious lives in soil and water, then we expect those lineages to be more ancient than those from animals. There is a comprehensive and recent study on this topic for *B*. *bronchiseptica*, which is a bacterium in the gram-negative Betaproteobacteria [35]. It causes respiratory infections in some species of mammals and is closely related to *B*. *pertussis*, which causes whooping cough in humans, accounting for about 89,000 deaths worldwide in 2008, according to the World Health Organization.

Soumana et al. constructed a phylogeny of *Bordetella* strains collected from environmental sources and from animals [36]. To do this, Soumana et al. searched the National Center for Biotechnology Information (NCBI) database for 16s ribosomal RNA sequence matches to several species of *Bordetella* and tied what they found to the sequence sources [36]. A neighbor-joining tree based on the 16S rRNA sequences indicated that environmental isolates were basal, as predicted [36].

This is not the only interesting thing about *Bordetella*. Most studies of amoeba–bacteria interactions take advantage of the similarities between amoebas and macrophages that are attributable to both having phagocytic activity [12,24]. While there are powerful advantages to using amoebas instead of animals as experimental hosts for bacteria, environmental amoebas generally live at much cooler temperatures (~21°C) than macrophages inside the human body (~37°C).

*B*. *bronchiseptica* has a two-component signal transduction system called BvgAS that regulates two distinct phases, the virulent Bvg+ phase and the avirulent Bvg− phase [37]. These systems operate differently at low and high temperatures [35]. At a higher temperature, virulence in the mammal host is regulated by Bvg+, which controls expression of over 100 genes [35]. At cooler temperatures, an equally large set of genes is activated in the Bvg− state. The latter genes allow growth at lower nutrient concentrations and turn on flagellar movement [35]. It turns out that the Bvg− state is what allows *B*. *bronchiseptica* to survive inside soil amoebas, including in the lab amoeba *D*. *discoideum* [35].

*B*. *bronchiseptica* remained present and alive after an hour when added to a culture of *D*. *discoideum* with the antibiotic gentamicin. By contrast, *B*. *bronchiseptica* could not survive an hour in the absence of *D*. *discoideum* with the same antibiotic. A standard food bacterium given to *D*. *discoideum* (namely, *Klebsiella pneumoniae*) was not present after an hour in either case, while the *B*. *bronchiseptica* bacteria were protected inside the amoebas. This result was confirmed with a similar experiment allowing *B*. *bronchiseptica* to invade another amoeba species distantly related to *D*. *discoideum*, *A*. *castellanii*.

When *D*. *discoideum* went through the social stage, *B*. *bronchiseptica* came right along, though outside the spores, which made it vulnerable at this stage to antibiotics (Fig 1B, purple). Not only did *B*. *bronchiseptica* bacteria survive in the fruiting bodies, but when the fruiting bodies were diluted 10-fold and replated on a new lawn of food, *B*. *bronchiseptica* proliferated right along with *D*. *discoideum*. This success of proliferation and survival in amoebas is due to the expression of the Bvg− system, something the authors demonstrated by showing how many fewer cells of a clone locked in the Bvg+ stage proliferated compared to either wild type or a clone locked in the Bvg− stage [35]. The authors further demonstrated that after passaging through spores of *D*. *discoideum*, the *B*. *bronchiseptica* were able to infect mouse respiratory tracts [35].

*Bordetella* is an ancient genus of bacteria that probably attacked environmental amoebas first but now also causes respiratory illness in mammals; this genus includes *B*. *pertussis*, which attacks only humans and is unable to survive in the environment [36].

Nevertheless, questions remain. Is *B*. *bronchiseptica* found in wild strains of *D*. *discoideum* or other species of *Dictyostelium*? Do other bacteria that invade both amoebas and animals have different sets of genes to adapt to both? Furthermore, a comprehensive survey of bacteria found in wild amoebas awaits future studies. Perhaps most insightful will be further discoveries of bacterial sequences in sequenced amoeba genomes.

## Conclusions

As McFall-Ngai and coauthors so nicely put it, animals evolved in a world that already contained billions of bacteria, archaea, and amoebas [38]. Thus, it is no surprise that some bacterial pathogens of humans and other mammals not only came from ancestors that attacked amoebas but often retained that ability over evolutionary time. These new and exciting results tell the detailed story of how a bacterium can exploit the social cycle of an amoeba and completely change the virulence genes it deploys according to whether it is attacking a hot mammal or a chilly amoeba. This example is likely to be only the first of many careful studies that reveal exactly how bacteria pull off these tricks.

## Abbreviations

LCV: *Legionella*-containing vacuole
NCBI: National Center for Biotechnology Information
T2SS: type II secretion system
T4SS: type IV secretion system
